# MRI versus histopathology in EMVI detection for rectal cancer: prognostic relevance and survival outcomes

**DOI:** 10.1007/s00330-026-12658-6

**Published:** 2026-06-10

**Authors:** Aida Kapic Lunder, Sebastian Meltzer, Elin Agathe Frøyen, Lars Gustav Lyckander, Ole Jacob Grandal, Anne Hansen Ree, Kathrine Røe Redalen, Anne Negård

**Affiliations:** 1 Department of Diagnostic Imaging, Akershus University Hospital, Lørenskog, Norway; 2 Department of Oncology, Akershus University Hospital, Lørenskog, Norway; 3 Department of Pathology, Akershus University Hospital, Lørenskog, Norway; 4 Department of Radiology, Diakonhjemmet Hospital, Oslo, Norway; 5 Institute of Clinical Medicine, University of Oslo, Oslo, Norway; 6 Department of Physics, Norwegian University of Science and Technology, Trondheim, Norway

**Keywords:** EMVI, Prognostic, Prospective, Rectal cancer, Survival

## Abstract

**Objectives:**

To evaluate the diagnostic accuracy of magnetic resonance imaging (MRI) compared to histopathology for detecting extramural venous invasion (EMVI) in rectal cancer, and to assess their prognostic value for long-term recurrence-free survival (RFS) and overall survival (OS).

**Materials and methods:**

In a prospective cohort of 150 patients with biopsy-confirmed rectal adenocarcinoma, EMVI was assessed via high-resolution 1.5-T MRI and histopathological examination. Interobserver agreement, clinical correlations, and survival outcomes were analyzed. Kaplan–Meier and Cox regression analyses were used to evaluate recurrence-free survival (RFS) and overall survival (OS).

**Results:**

mrEMVI was detected in 52.7% of patients and showed good interobserver agreement (κ = 0.68). pEMVI was confirmed in 45.9% of surgical cases, with fair agreement with mrEMVI (κ = 0.40). mrEMVI was significantly associated with advanced tumor stage, larger tumor volume, synchronous metastases, and elevated CEA levels. mrEMVI positivity predicted poorer RFS (HR 5.22, *p* < 0.001) and OS (HR 3.40, *p* = 0.01), while pEMVI showed no significant prognostic value.

**Conclusion:**

mrEMVI is a more reliable imaging biomarker strongly correlated with long-term oncologic events compared to histopathological EMVI.

**Key Points:**

***Question*** *Reliable preoperative markers are lacking for accurate EMVI detection in rectal cancer, creating uncertainty in risk stratification and treatment decisions due to inconsistent MRI–pathology concordance.*

***Findings*** *mrEMVI showed strong prognostic value for recurrence and survival, whereas pEMVI did not, and MRI offered higher reliability and reproducibility than histopathology.*

***Clinical relevance*** *Identifying mrEMVI preoperatively enables more accurate risk stratification, helping clinicians tailor treatment intensity and potentially improve survival, while highlighting MRI as a more reliable tool than pathology for guiding management decisions in rectal cancer.*

**Graphical Abstract:**

**Figure:**
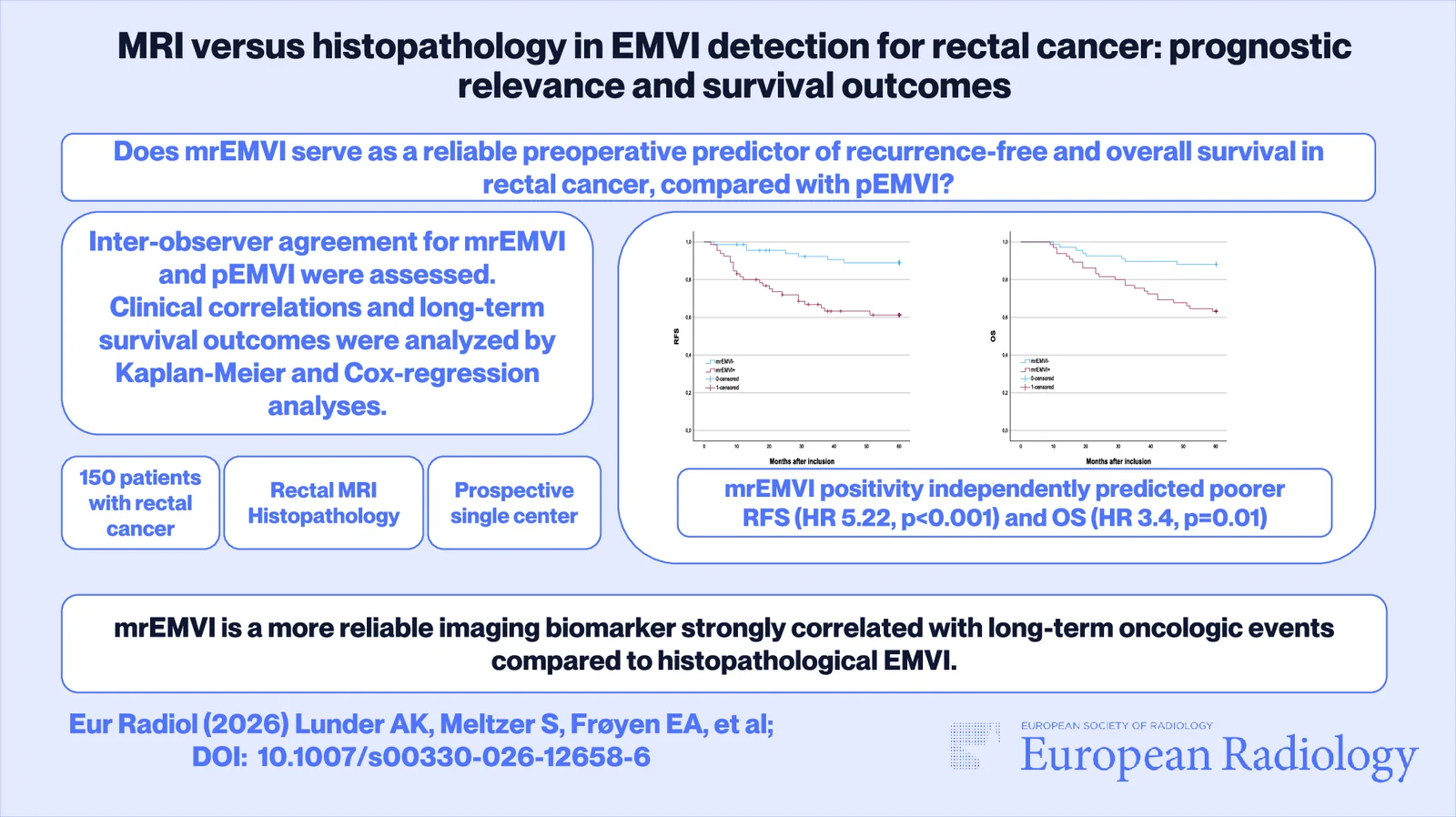

## Introduction

Despite advancements in treatment options for rectal cancer, the 5-year survival rate remains approximately 70%, with nearly one-third of patients facing a fatal outcome [1]. Current therapeutic modalities include surgery, chemotherapy, radiotherapy, or a combination of these. Until recently, neoadjuvant chemoradiotherapy (CRT) was considered the standard treatment for locally advanced disease in most Western countries [2]. Accurate preoperative diagnosis is crucial to guiding treatment decisions effectively and to improving future patient outcomes. Choosing the appropriate treatment requires careful consideration to prevent under-treatment, which can lead to local recurrence and metastasis, and over-treatment, which may result in unnecessary complications and an impaired quality of life. Consequently, there is a pressing demand for reliable prognostic markers to inform clinical decision-making.

MRI has become a vital tool in the preoperative assessment of rectal cancer, significantly enhancing tumor, node, and metastasis (TNM) staging accuracy. Among the important MRI findings, EMVI has been recognized as a significant prognostic factor [3, 4]. Patients with mrEMVI positivity are at an increased risk of visceral metastases, particularly to the liver. The tumor cells in the vasculature beyond the muscularis propria of the bowel wall drain via the portal venous system directly to the liver [5]. Although anatomical MRI can detect EMVI, the reproducibility of mrEMVI remains inconsistent, and its impact on disease recurrence and survival warrants further investigation.

While the relationship between pathologically diagnosed EMVI (pEMVI) and metastatic disease is well-established [6], its prevalence can vary significantly across different populations [7], and it may be underreported due to imprecise definitions and analytical methods [8]. Recent advancements, such as the use of elastin stains [9], aim to address these diagnostic challenges. However, the comparative accuracy of mrEMVI versus pEMVI is still unclear, leading to concerns about the reliability of EMVI assessments.

This study aims to evaluate the presence and inter-reader agreement of EMVI in rectal cancer using MRI and histopathology that includes elastin stains. We also investigate the prognostic value of both mrEMVI and pEMVI among other clinical and radiological factors concerning 5-year recurrence-free survival. By comparing the reliability of EMVI detection through pathology and MRI, this study seeks to highlight the prognostic importance of EMVI regardless of the assessment method.

## Materials and methods

### Study population

**Fig. 1:**
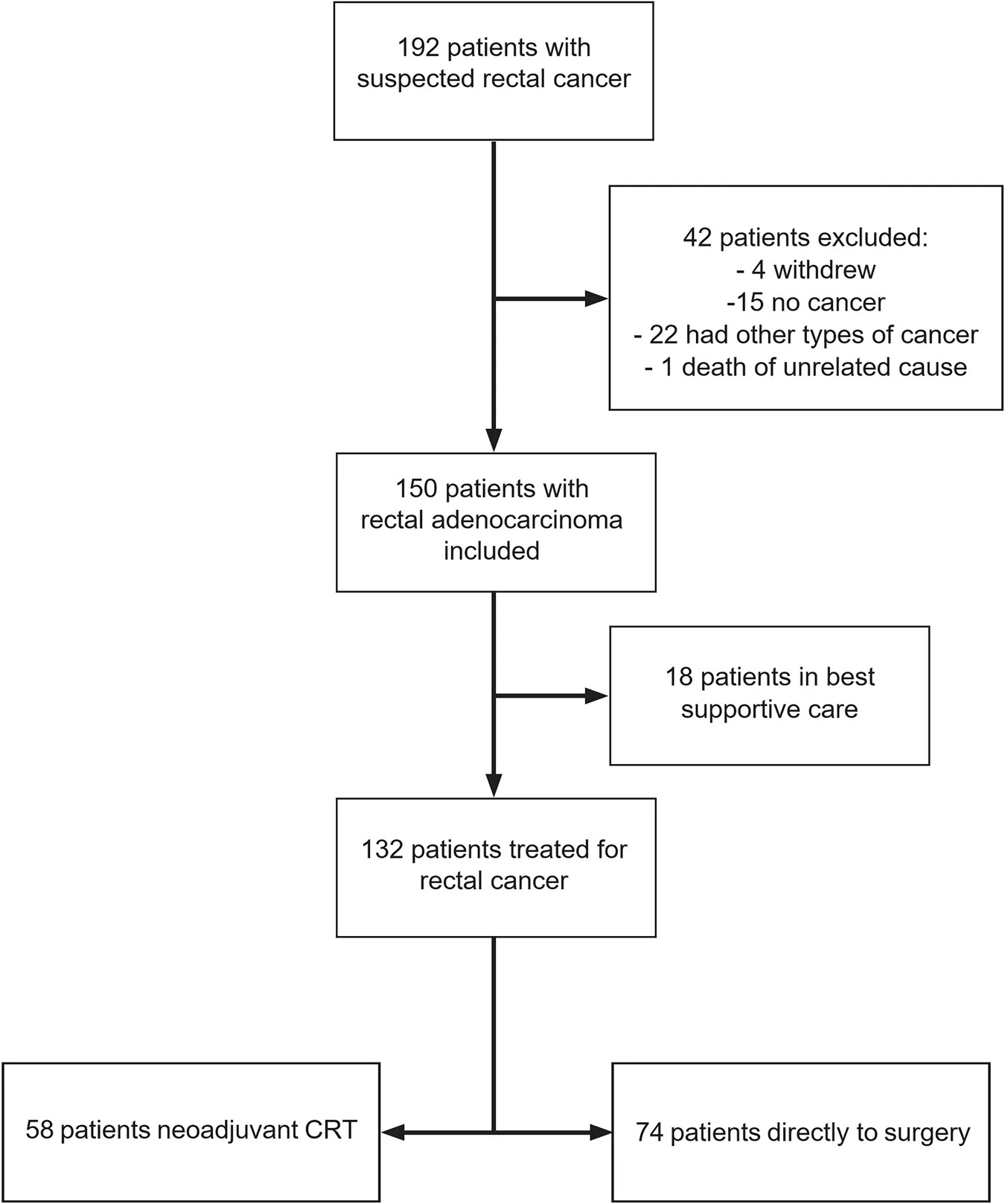
Flowchart

Between October 28, 2013, and December 19, 2017, 192 patients referred to our hospital with suspected rectal cancer consented to participate in a prospective biomarker study (OxyTarget Study, ClinicalTrials.gov: NCT01816607). All patients included in this analysis were drawn from the cohort. Patients underwent standard diagnostic procedures, including colonoscopy, rigid rectoscopy, computed tomography (CT) of the thorax, abdomen, and pelvic cavity, and rectal MRI. Of these, 150 patients with biopsy-proven primary rectal or rectosigmoid adenocarcinoma, located within 15 cm of the anal verge (as identified by rigid rectoscopy), were included. These patients were all unique to this specific analysis of EMVI, with no overlap or repeated entries. Clinical data were obtained from the hospital’s electronic patient record system. Blood samples were collected at the time of patient enrollment, shortly before the scheduled pelvic MRI. A flowchart of the study design is shown in Fig. 1.

### Imaging

MRI examinations were conducted using a 1.5-T Philips Achieva dStream MRI unit, with both posterior and anterior coils (44-channel phased-array body coil posterior, 32-channel anterior coil). The slice thickness was set to 3 mm with a gap of 0.3 mm (i.e., 10% gap), resulting in 0.6 × 0.6 mm voxel size with isotropic 1 mm resolution for 3D T2-weighted imaging. The echo time (TE) was 90 ms, and the repetition time (TR) was 3000 ms. No bowel preparation was required, but patients received Buscopan (20 mg IV) and Glucagon (1 mg intramuscularly) to reduce colonic motility.

### Image analysis

**Fig. 2:**
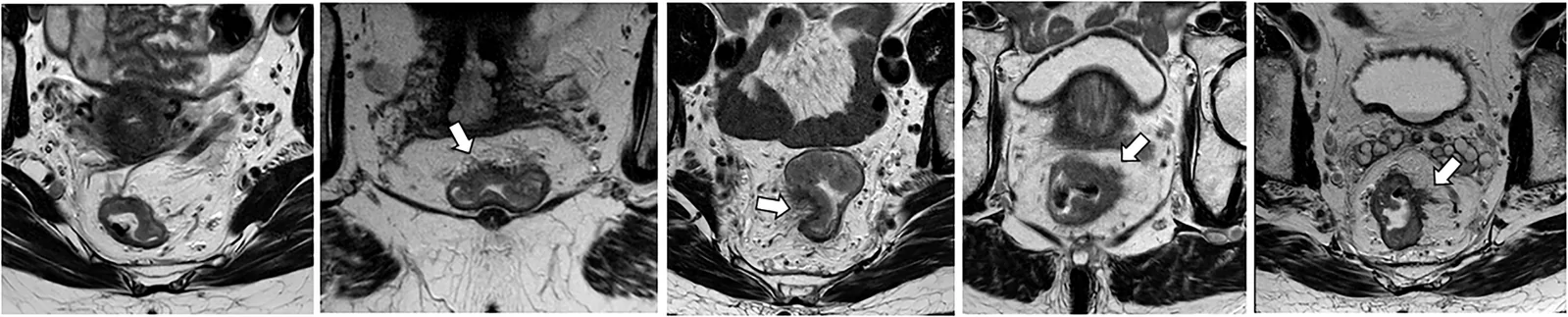
EMVI scoring system for MRI. Left to right: score 0—no EMVI; score 1—minimal extramural stranding, probably no EMVI (white arrow); score 2—stranding in proximity of vessels, no tumor signal, equivocal (white arrow); score 3—vessel expansion, probably present EMVI (white arrow); score 4—tumor signal, vessel expansion and irregular vessel contour, definitely present EMVI (white arrow). EMVI status is negative for scores 0–2 and positive for scores 3–4

Two consultant radiologists (O.J.G. and E.A.F.) independently reviewed all MRI images, blinded to prior reports and patient outcomes. Reader 1 had 10 years of experience in gastrointestinal radiology with a specialization in rectal MRI, while Reader 2 had 2 years of experience in the same field. EMVI was defined as irregular, serpentine tumor extensions originating from the primary tumor on T2-weighted images. A 5-point grading system was utilized to evaluate EMVI [3], with representative MRI images illustrating the grading system shown in Fig. 2. To ensure consistent interpretation of the rating criteria, both radiologists initially reviewed 10 unrelated cases together for standardization. Tumor volume was manually contoured on T2-weighted MR images by the radiologists. It was calculated by multiplying the tumor area in each slice by the sum of the slice thickness and inter-slice gap.

### Pathology

**Fig. 3:**
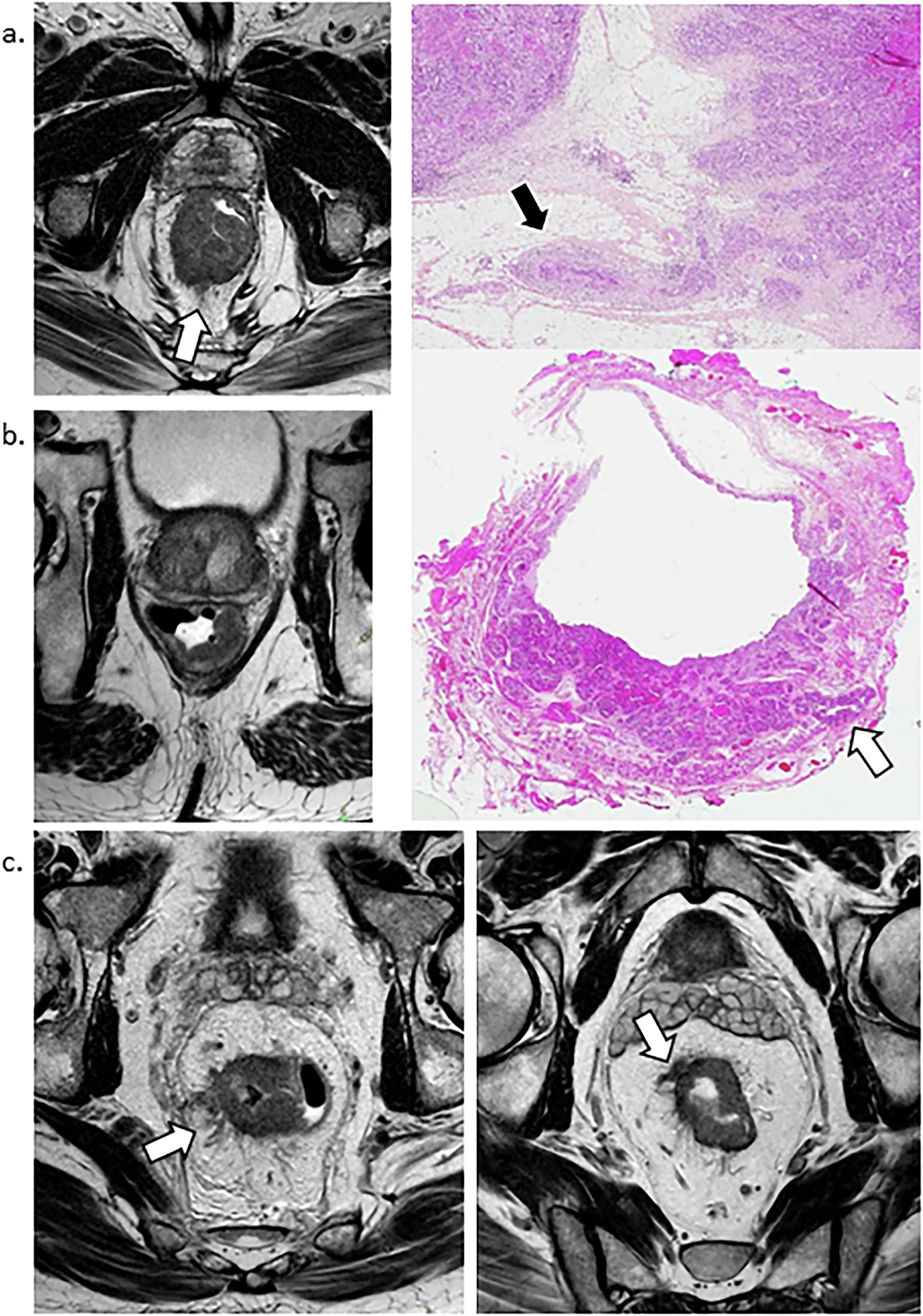
Transverse oblique T2-weighted MRI and corresponding H&E-stained pathological images showing EMVI. **a** Tumor signal in vessel (left side, white arrow) and tumor within a vein (right side, × 18 magnification, black arrow), **b** no apparent mrEmvi (left) and tumor in vein (white arrow). **c** Vessel expansion and irregular contour (white arrows) in both images, no corresponding pEMVI detected (not shown)

A consultant pathologist with 10 years of experience in rectal cancer pathology re-examined the histopathology blocks to assess the presence of EMVI using hematoxylin-phloxine-saffron (HPS) staining. Verhoff’s elastic staining was applied when the EMVI status was uncertain. All pathology evaluations were conducted independently, without access to the MRI results. Specimens were classified as EMVI-positive or EMVI-negative. In EMVI-negative cases, further evaluation was conducted for intramural vascular invasion or submucosal invasion, in accordance with The Royal College of Pathologists’ standards [10]. EMVI was identified when the tumor was located within an endothelium-lined space, surrounded by a muscle coat or containing red blood cells. In addition, two key imaging features were considered indicative of EMVI: the “orphan artery” sign, characterized by a rounded or elongated tumor profile adjacent to an artery without an accompanying vein; and the “protruding tongue” sign, defined as a smooth-bordered protrusion of the tumor into perirectal fat near an artery (Fig. 3).

### Statistics

Independent samples *t*-test or the Wilcoxon rank-sum test was used to compare continuous data, while chi-square and Fisher’s exact tests were employed for categorical data comparisons. To identify specific group differences within the multi-category variable, pairwise comparisons were conducted using the Bonferroni correction. Kappa (κ) statistics were used to assess agreement on EMVI presence (scores 3–4) versus absence (scores 0–2) between the radiologists and the pathologist. Kappa values were interpreted as follows: < 0.20 = poor agreement, 0.21–0.40 = fair, 0.41–0.60 = moderate, 0.61–0.80 = good, and > 0.80 = excellent agreement. Regression analyses were performed to examine the independent associations between EMVI and clinical variables. Survival analysis was conducted using Kaplan–Meier plots and Cox regression analysis. Overall survival (OS) was defined as the time from surgery to death, while relapse-free survival (RFS) was defined as the time from surgery to any local or distant recurrence. Proportional hazards assumptions were evaluated using Schoenfeld residual–based tests for all Cox models, and model discrimination was assessed using Harrell’s C‑index. All statistical analyses were performed using SPSS version 27 and STATA version 18.0, with a significance threshold set at *p* < 0.05.

### Ethical considerations

The study protocol was approved by the Regional Committee for Medical and Health Research Ethics in South Eastern Norway (REK 2013/152) and the institutional review board. Informed written consent was obtained from all participants.

## Results

A total of 150 patients with rectal adenocarcinoma were included in the study, with demographic details summarized in Table 1. The cohort exhibited a male predominance, accounting for 64.7% of participants, with a mean age of 64.4 years (SD 10.8). Tumor location was primarily in the mid and upper rectum, comprising 76% of cases. Mucinous adenocarcinomas constituted 8.7% of the tumors.

**Table 1:** Study demographics

Characteristics/group	Total (*N* = 150)
Sex, *n* (%)	
Female	53 (35.3)
Male	97 (64.7)
Age, years	
Mean (SD)	64.4 (10.8)
BMI, kg/m^2^	
Mean (SD)	26.0 (4.3)
Tumor location, *n* (%)	
Low (< 5 cm)	36 (24)
Mid (5–10 cm)	57 (38)
High (> 10 cm)	57 (38)
Mean tumor volume (*n* = 126) cm^2^ (SD)	30.3 (28.5)
Median MRF distance (*n* = 115) mm (range)	6.4 (0–50)
Type of tumor	
Adenocarcinoma, *n* (%)	137 (91.3)
Mucinous tumor, *n* (%)	13 (8.7)
mrT staging (7th ed.), *n* (%)	
T1/T2	33 (22)
T3	69 (46)
T4	48 (32)
T4a	20
T4b	28
mrN staging (7th ed.), *n* (%)	
N0	69 (46)
N1	50 (33.3)
N2	31 (20.7)
Synchronous metastasis, *n* (%)	31 (20.7)
Liver	22 (71.0)
Lung	2 (6.4)
Lymph nodes	1 (3.2)
Multiple sites	6 (19.4)
Disease stage (ACR 2016), *n* (%)	
Stage I	30 (20)
Stage II	36 (24)
Stage III	52 (34.7)
Stage IV	31 (20.7)
CEA, mg/L (*n* = 145) (SD)	25.3 (90.9)

*MRF* mesorectal fascia, *CEA* carcinoembryonic antigen, *ACR* American College of Radiology

Most patients were classified as having ACR disease stage III (*n* = 52, 34.7%). The distribution of the remaining stages was relatively balanced, with approximately 20% to 24% of patients in each group. Notably, 20.7% of patients (*n* = 31) presented synchronous metastases at diagnosis, predominantly to the liver (*n* = 26, 83.9%). In terms of treatment modalities, half of the patients (*n* = 74, 49.3%) underwent surgery alone. A significant portion, 38.7% (*n* = 58), received neoadjuvant CRT prior to surgery, while 12% (*n* = 18) were deemed inoperable and subsequently offered palliative tumor-directed therapy. The median follow-up period for the entire cohort was 60 months (range 2–60 months).

### mrEMVI interobserver agreement

The interobserver agreement for the presence of mrEMVI was found to be good (κ = 0.68; Supplementary Data S1). The overall agreement among radiologists reached 0.84, with a positive agreement of 0.83 and a negative agreement of 0.85. By consensus, 79 patients (52.7%) were diagnosed with EMVI. Furthermore, the presence of EMVI on MRI was significantly associated with low (< 5 cm) and high (> 10 cm) tumor location (*p* = 0.005), increasing tumor volume (*p* = 0.002), advanced ACR 2016 disease stage (*p* < 0.001), higher MRI T-stage (*p* < 0.001) and N-stage (< 0.001), as well as synchronous metastasis at diagnosis (*p* = 0.008), and elevated carcinoembryonic antigen (CEA) levels (*p* = 0.001) (Table 2).

**Table 2:** Demographic and clinical characteristics according to mrEMVI and pEMVI status

Variables	mrEMVI status, *n* = 150			pEMVI status, *n* = 74		
	mrEMVI+	mrEMVI−	*p*-value	pEMVI+	pEMVI−	*p*-value
Sex, *n* (%)						
Female	25 (31.6)	28 (39.4)		10 (29.4)	20 (50)	
Male	54 (68.4)	43 (60.6)	0.32	24 (70.6)	20 (50)	0.07
Mean age, years (SD)	64.3 (10.6)	64.4 (10.9)	0.63	67.2 (10.2)	63.0 (11.2)	0.09
Mean BMI, kg/m^2^ (SD)	26.2 (4.4)	25.8 (4.2)	0.62	25.8 (3.6)	26.8 (4.4)	0.25
Tumor location, *n* (%)						
Low (< 5 cm)	11 (13.9)	25 (35.2)		3 (8.8)	7 (17.5)	
Mid (5–10 cm)	31 (39.2)	26 (36.6)		10 (29.4)	18 (45)	
High (> 10 cm)	37 (46.8)	20 (28.2)	*0.005*	21 (61.8)	15 (37.5)	0.11
Mean tumor volume, cm^2^ (SD) (*n* = 132)	39.8 (33.3)	19.6 (15.5)	*0.002*	24.6 (17.6)	18.3 (13.7)	0.14
Median MRF distance, mm (SD) (*n* = 132)	5.3 (7)	7.7 (11.1)	0.05	7.6 (7.2)	13.1 (13.1)	*0.046*
Mucinous tumor, *n* (%)	7 (10.3)	6 (9.8)	0.93	2 (5.9)	3 (7.5)	1.00
mrT staging (7th ed.), *n* (%)						
T1/T2	1 (1.3)	32 (45.1)		8 (23.5)	21 (52.5)	
T3	40 (50.6)	29 (40.8)		17 (50)	16 (40)	
T4	38 (48.1)	10 (14.1)	*< 0.001*	9 (26.5)	3 (7.5)	*0.023*
mrN staging (7th ed.), *n* (%)						
N0	19 (24.1)	60 (70.4)		16 (47.1)	33 (82.5)	
N1	30 (38)	20 (28.6)		15 (44.1)	6 (15)	
N2	30 (38)	1 (1.4)	*< 0.001*	3 (8.8)	1 (2.5)	*0.004*
Synchronous metastasis, *n* (%)	23 (29.1)	8 (11.3)	*0.008*	7 (21.2)	6 (15.8)	0.56
Disease stage (ACR), *n* (%)						
Stage I	1 (1.3)	29 (40.8)		8 (23.5)	21 (52.5)	
Stage II	16 (20.3)	20 (28.2)		6 (17.6)	12 (30)	
Stage III	35 (44.3)	17 (23.9)		15 (44.1)	6 (15)	
Stage IV	27 (34.2)	4 (5.6)	*< 0.001*	5 (14.7)	1 (2.5)	*0.003*
CEA, mg/L (SD)	40 (118.5)	9 (38.5)	*0.001*	5.3 (10)	5 (4.8)	0.32

*n* = number

*p*-value in italic are significant values, i.e. *p* < 0-05.

Among the 150 patients, 74 underwent curative surgery without neoadjuvant treatment and had available pathological specimens. Of these, 34 patients (45.9%) were diagnosed with pathologically confirmed EMVI (pEMVI). The implementation of elastin staining increased the prevalence of pEMVI from 30 to 34 patients. The presence of pEMVI was significantly associated with lower median mesorectal fascia (MRF) distance (*p* = 0.046), higher MRI T-stage (*p* = 0.02), N-stage (*p* = 0.004) and ACR 2016 stage (*p* = 0.003) (Table 2). The interobserver agreement between radiologist consensus data and pathology findings was fair (κ = 0.40) for the presence of EMVI (Supplementary Data S2). The observed agreement was 0.70, with a positive agreement of 0.67 and a negative agreement of 0.75. A significant association was observed between pEMVI and mrEMVI scores (*p* = 0.006), reflected by an over‑representation of pEMVI‑negative cases in mrEMVI category 0 and a higher proportion of pEMVI‑positive cases in mrEMVI category 4, indicating alignment at the extremes of the scoring spectrum (Supplementary Data S3).

### Survival

Among the 132 patients treated with curative intent, 117 (88.6%) were metastasis-free at baseline. During the follow-up period, 23 patients developed metastases, with the following distribution: 12 patients had liver-only metastases, 3 had lung-only metastases, 4 had multiple metastatic sites, 2 developed peritoneal carcinomatosis, 1 developed bone metastasis, and 1 had lymph node involvement. The cases with multiple metastatic sites included 3 patients with lung metastases, 2 with liver metastases, 1 with bone metastasis, and 1 with both lymph node and brain metastases. In total, 33 patients (28.2%) died during the follow-up period.

**Fig. 4:**
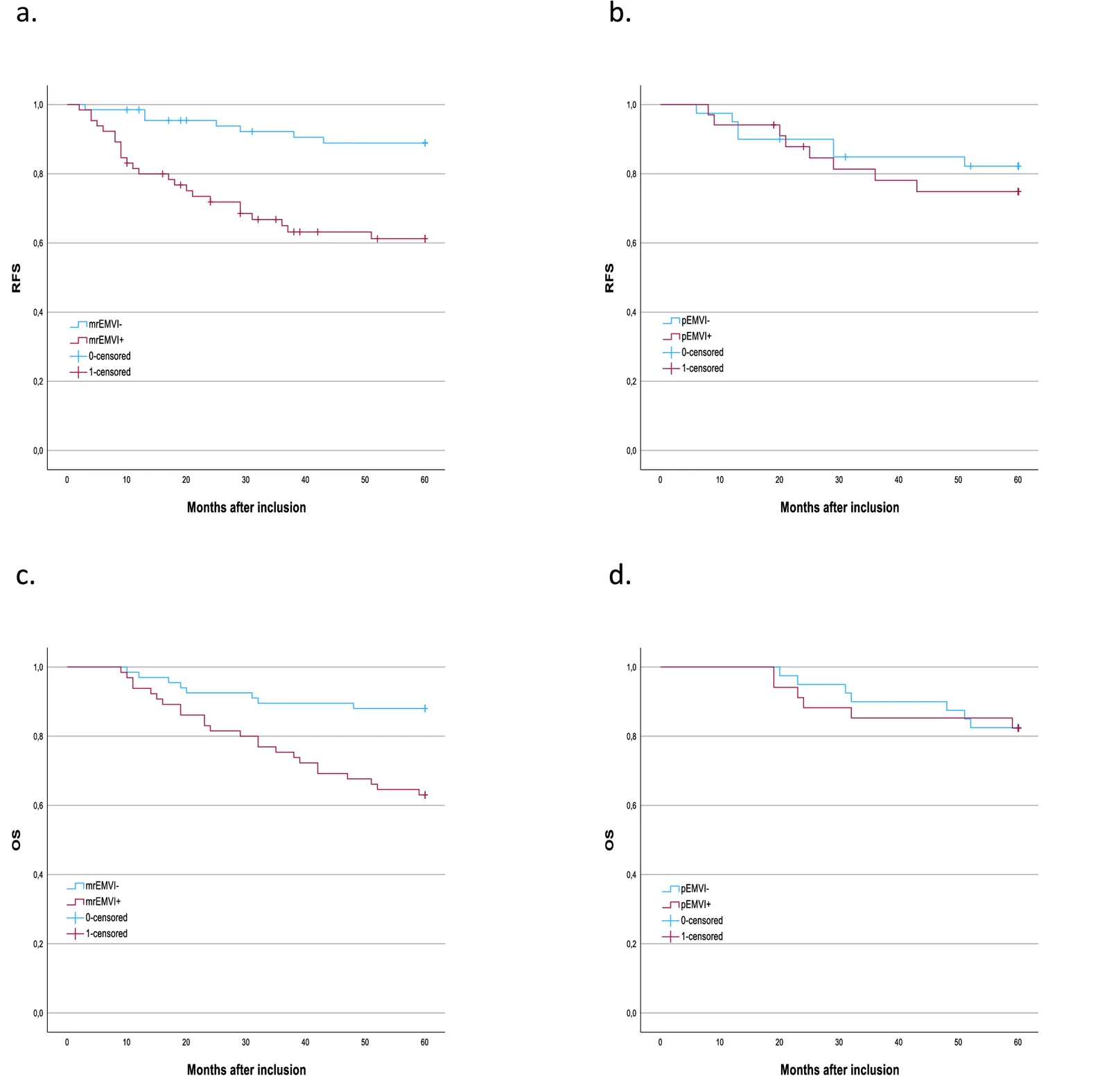
Kaplan–Meier survival curves for RFS and OS based on **a**, **b** mrEMVI and **c**, **d** pEMVI status

Figure 4 presents Kaplan–Meier survival curves illustrating the relationship between EMVI status and RFS and OS. In Fig. 4a, b, patients who tested positive for mrEMVI showed significantly poorer outcomes in both RFS (*p* < 0.001) and OS (*p* = 0.001) compared to those who were negative for mrEMVI. In contrast, Fig. 4c, d shows that there was no significant difference in either RFS (*p* = 0.48) or OS (*p* = 0.95) between patients who were pEMVI positive and those who were negative for pEMVI. In the surgery-only subgroup (*n* = 74), recurrence occurred in 8/34 (23.5%) pEMVI+ patients and 7/40 (17.5%) pEMVI− patients (RR 1.34, 95% CI 0.54–3.33; *p* = 0.57). Death occurred in 6/34 (17.6%) pEMVI+ and 7/40 (17.5%) pEMVI− patients (RR 1.01, 95% CI 0.37–2.71; *p* = 1.00).

### Univariate and multivariate analyses

Table 3 presents univariate and multivariate analyses for RFS. As for univariate analysis, both mrEMVI positivity (HR 4.33, 95% CI 1.86–10.07, *p* < 0.001) and a shorter MRF distance (HR 0.93, 95% CI 0.85–0.99, *p* = 0.04) were significantly associated with poorer RFS. Both MrEMVI positivity (HR 5.22, 95% CI 1.96–13.94, *p* < 0.001) and shorter MRF distance (HR 0.92, 95% CI 0.85–0.99, *p* = 0.03) remained independent predictors of adverse outcome in the multivariate analysis.

**Table 3:** Univariate and multivariate analyses for RFS (relapse-free survival)

Variable	RFS, *n* = 132 Univariate		RFS, *n* = 132 Multivariate	
	HR (95% CI)	*p*-value	HR (95% CI)	*p*-value
Age	1.02 (0.99–1.06)	0.21	1.03 (0.99–1.07)	0.16
Sex	1.72 (0.77–3.85)	0.19	1.67 (0.67–4.16)	0.27
BMI	1.06 (0.98–1.16)	0.17		
Disease stage (ACR 2016)				
1	2.07 (0.66–6.51)	0.21		
2	2.13 (0.68–6.61)	0.19		
3	2.48 (0.62–9.96)	0.20		
4	0	0.98		
CEA	1 (0.96–1.02)	0.27		
Tumor volume	1.00 (0.99–1.02)	0.08		
MRF distance	0.93 (0.86–0.99)	0.04	0.92 (0.85–0.99)	0.03
mrEMVI	4.33 (1.86–10.07)	< 0.001	5.22 (1.96–13.94)	< 0.001

Table 4 presents univariate and multivariate analyses for OS. Significant factors for poorer OS included higher CEA level (1.01, 95% CI 1–1.02, *p* = 0.004), shorter MRF distance (HR 0.92, 95% CI 0.86–0.99, *p* = 0.03), and mrEMVI positivity (HR 3.50, 95% CI 1.57–7.79, *p* = 0.002), shown in Table 4. mrEMVI positivity (HR 3.40, 95% CI 1.34–8.61, *p* = 0.01) and shorter MRF distance (HR 0.92, 95% CI 0.86–0.99, *p* = 0.03) remained independent predictors of death in the multivariate analysis.

**Table 4:** Univariate and multivariate analyses for OS (overall survival)

Variable	OS Univariate		OS Multivariate	
	HR (95% CI)	*p*-value	HR (95% CI)	*p*-value
Age	1.01 (0.98–1.04)	0.59	1.01 (0.98–1.06)	0.45
Sex	0.93 (0.64–1.34)	0.69	0.97 (0.44–2.13)	0.94
BMI	1.07 (0.99–1.16)	0.99		
Disease stage (ACR 2016)				
1	0.84 (0.26–2.76)	0.78		
2	1.78 (0.63–4.96)	0.28		
3	3.78 (1.24–11.57)	0.02		
4	0	0.98		
CEA	1.01 (1.00–1.02)	0.004		
Tumor volume	1.00 (0.99–1.01)	0.87		
MRF distance	0.92 (0.86–0.99)	0.03	0.92 (0.86–0.99)	0.03
mrEMVI	3.50 (1.57–7.79)	0.002	3.40 (1.34–8.61)	0.01

The proportional hazards assumption was met for the RFS model (*p* = 0.08). For OS, a violation was observed due to the continuous MRF distance variable (*p* = 0.01); removal of this covariate resolved the issue (*p* = 0.17). Model discrimination was good (C-index RFS 0.76; OS 0.69).

## Discussion

This study evaluated the prognostic significance and inter-reader agreement of EMVI detection in rectal cancer using MRI and histopathology that included elastin staining. Patients who were mrEMVI positive have about a 5-fold higher hazard of recurrence and a 3.4 times higher hazard of death. This large effect size highlights the clinical relevance of mrEMVI as a preoperative biomarker. A threatened MRF was a strong adverse feature, and increasing clearance was associated with better relapse outcomes.

The good interobserver agreement for mrEMVI detection (κ = 0.68) suggests that MRI is a reproducible tool for identifying EMVI, aligning with previous studies reporting moderate to good agreement [11]. Although pEMVI correlated with adverse staging features, such as shorter MRF distance and higher T/N-stage, it did not demonstrate prognostic significance in this cohort. This may reflect limited power in the pathology subset and potential under-detection of EMVI on histopathology despite elastin staining, supported by only fair agreement between mrEMVI and pEMVI (κ = 0.40). These findings highlight challenges in correlating imaging findings with histopathological results. A study from 2024 [11] reported similar data on mrEMVI and pEMVI agreement (κ = 0.40), consistent with our findings. Several factors may explain the discrepancy. MRI evaluates the entire tumor and mesorectal vasculature in vivo, while pathology is limited by sampling and tissue shrinkage during fixation. Sampling variability, anatomical complexity of rectal vasculature and the inability to trace vein courses often lead to underreported EMVI in histopathology. Although interobserver variability for pEMVI is known to be moderate at best, we believe the more prominent issue is underreporting of EMVI in routine pathology. In contrast, mrEMVI may reflect a broader biological phenotype of extramural vascular spread that is not always captured in a single histological section. This could explain why mrEMVI aligns more consistently with oncologic outcomes in our study.

The superior prognostic performance of mrEMVI compared to pEMVI is consistent with the literature citing difficulties in histopathological assessment, particularly after neoadjuvant therapy, due to fibrosis and sampling limitations [12]. While histopathology is often considered the gold standard, its imperfections can underestimate the accuracy of MRI and other diagnostic tools. mrEMVI may capture prognostically relevant venous invasion more consistently in this cohort, although this must be interpreted with caution, given the smaller pathology subgroup and potential sampling limitations.

A meta-analysis by Shin and co-workers [13] estimated mrEMVI prevalence at 35.7%, significantly lower than our observed rate of 52.7%. This discrepancy reflects differences in patient cohorts, with our study including a higher proportion of locally advanced-stage rectal cancer cases. In our cohort, 59% of patients had regional rectal cancer (stage 2 and 3), compared to 36% in the general American population, as reported by the Surveillance, Epidemiology, and End Results (SEER) program and the American Cancer Society [14]. The association of EMVI with advanced T and N stages, larger tumor volumes, and elevated CEA levels underscores its role in systemic disease dissemination and prognosis. A recent meta-analysis [15] identified a pooled risk ratio of 4.11 for synchronous metastases, a significant correlation between mrEMVI and worse OS (HR = 1.68) as well as disease-free survival (HR = 2.30) in mrEMVI-positive patients, findings that closely parallel our results. Conversely, the absence of a survival association for pEMVI likely reflects both limited sensitivity and inadequate statistical power. It is also worth considering that there may be a threshold level of EMVI, such as the extent or volume of venous invasion, required before the prognostic impact becomes apparent. A plausible interpretation is that mrEMVI better captures macroscopic extramural venous invasion linked to systemic spread than standard block‑based pathology. Further studies are needed to define such thresholds to refine prognostication.

Several advanced MRI techniques show promise for improving EMVI assessment beyond conventional T2‑weighted imaging. Diffusion‑weighted imaging (DWI) offers added diagnostic value, particularly after chemoradiotherapy [16], and newer implementations, such as reduced field‑of‑view DWI and high b‑value synthetic DWI, enhance lesion detection. Advanced diffusion models like IVIM and perfusion‑based techniques such as DCE‑MRI may provide complementary physiologic information, and radiomics approaches built from DCE features have demonstrated superior predictive performance compared with traditional parameters [17]. These functional and quantitative methods may help address limitations of morphology‑based EMVI evaluation and reduce inter‑observer variability. Future work should prioritize optimized MRI protocols, standardized EMVI reporting, and integration of radiomics and artificial intelligence, supported by larger multicenter studies to validate these approaches and refine risk stratification.

To further reconcile disparities between imaging and pathology, future studies may benefit from whole‑mount histopathology, which enables complete circumferential evaluation of the mesorectum and precise spatial correlation with MRI. Whole‑mount processing could better capture focal or segmental EMVI, reduce sampling error, and provide a more appropriate reference standard for radiology–pathology matching.

The study’s strengths include a prospective study design, a well-defined cohort, standardized imaging protocols, and detailed histopathological assessment. However, the single-institution design and modest sample size may limit external validity. Our study was performed on a 1.5‑T scanner, and although ESGAR recommends both 1.5‑T and 3‑T systems [16], evidence shows that 3‑T MRI offers better rectal wall visualization [17] and would be expected to improve EMVI assessment. Our findings may therefore underestimate the true prognostic strength of mrEMVI, which could be more accurately evaluated using 3‑T imaging. The absence of a survival association for pEMVI should be interpreted cautiously, as the smaller pathology subset and limited number of events indicate insufficient power rather than true prognostic irrelevance. Moreover, the only fair concordance between mrEMVI and pEMVI underscores the likelihood of histopathological under‑detection and supports the complementary role of MRI.

In conclusion, our results underscore that mrEMVI provides a robust and reproducible prediction of 5-year RFS and OS. As a reliable preoperative imaging biomarker strongly correlated with oncologic events, mrEMVI offers greater clinical utility than postoperative histopathological EMVI in informing therapeutic decision-making and potentially improving oncologic outcomes.

## Supplementary information

ELECTRONIC SUPPLEMENTARY MATERIAL

## Abbreviations

CRT: Chemoradiotherapy
EMVI: Extramural venous invasion
OS: Overall survival
RFS: Recurrence-free survival
TNM: Tumor node metastasis
