# Supplementary material for: MRI versus histopathology in EMVI detection for rectal cancer: prognostic relevance and survival outcomes
Source: Eur Radiol. 2026 Jun 10;36(10):7974–84. doi: 10.1007/s00330-026-12658-6 (PMC13574916; doi:10.1007/s00330-026-12658-6)
Supplement: Supplementary file 1 [file 330_2026_12658_MOESM1_ESM.pdf]

MRI versus Histopathology in EMVI Detection for Rectal Cancer: Prognostic Relevance and Survival Outcomes

ELECTRONIC SUPPLEMENTARY MATERIAL

Table S1. Inter-observer agreement on mrEMVI status between two radiologists.

**Rad1/Rad2**	**mr-EMVI-**	**mr-EMVI+**	**Total**
**mr-EMVI-**	69	14	83
**mr-EMVI+**	10	57	67
**Total**	79	71	150

Table S2. Inter-observer agreement on EMVI status between mr-EMVI and p-EMVI.

	**mr-EMVI-**	**mr-EMVI+**	**Total**
**p-EMVI-**	30	8	38
**p-EMVI+**	13	20	33
**Total**	43	28	71

Table S3. Crosstabulation of pEMVI status by consensus mrEMVI by category 0-4.

A significant association was found between pEMVI and mrEMVI (Pearson $\chi^2 = 13.159$, df = 4, p = 0.011; Fisher–Freeman–Halton exact p = 0.006). Adjusted residuals showed significantly more pEMVI-negative cases in mrEMVI category 0 (not present) and more pEMVI-positive cases in mrEMVI category 4 (present).

**Consensus mrEMVI**	**pEMVI -**	**pEMVI+**	**Total**
**0- Not present**	18 (69,2%)<br>Adjusted Residual 2.0	8 (30.8%)<br>Adjusted Residual -2.0	26
**1- Probably not present**	1 (50%)	1 (50%)	2
**2- Equivocal**	11 (73.3%)	4 (26.7%)	15
**3- Probably present**	6 (37.5%)	10 (62.5)	16
**4- Present**	2 (16.7%)<br>Adjusted residual -2.8	10 (83.3%)<br>Adjusted residual 2.8	12
**Total**	38	33	71
